# Predictors of Long COVID‐19 Syndrome and Hospital Admissions Among COVID‐19‐Diagnosed Adult Patients Who Self‐Isolated at Home in KwaZulu‐Natal Province, South Africa

**DOI:** 10.1155/nrp/9317685

**Published:** 2026-05-06

**Authors:** Moshibudi Poncho Phafane, Alone Isabirye, Poovendhree Reddy

**Affiliations:** ^1^ Division of Public Health Surveillance and Response, National Institute for Communicable Diseases, Johannesburg, Gauteng, South Africa, nicd.ac.za; ^2^ KwaZulu-Natal Provincial Department of Health, Communicable Disease Control, Pietermaritzburg, KwaZulu-Natal, South Africa; ^3^ Department of Community Health Studies, Durban University of Technology, Durban, KwaZulu-Natal, South Africa, dut.ac.za

**Keywords:** hospitalisation, morbidity, one health, pandemics, post-acute COVID-19 syndrome, reinfection, resource allocation, SARS-CoV-2 variants

## Abstract

Long COVID‐19 (LC) syndrome is a complex systemic illness that is currently recognised to have a high morbidity rate and hospitalisations. The study analysed factors linked to LC in adults self‐isolated during COVID‐19 infection in KwaZulu‐Natal Province, South Africa, focusing on two densely populated districts (uMgungundlovu and eThekwini Metro Municipality). We employed a cross‐sectional study among individuals aged 18 years and above who self‐isolated at home. Demographic data, COVID‐19 vaccination status, and post‐COVID‐19 health symptoms were collected using a standardised questionnaire. The National Health and Nutrition Survey’s Physical Functioning Questionnaire was adapted to evaluate health and functional outcomes six months after a COVID‐19 diagnosis, addressing both physical and psychosocial symptoms during that timeframe. A modified Poisson regression model was used to determine the predictors of LC and hospitalisation. Of the 280 participants, 46% (*n* = 130) reported having at least one health‐related symptom, while 36% (*n* = 47) had ≥ five symptoms. Approximately half of the participants (50%, *n* = 139) had at least one hospital admission following infection due to persistent symptoms. Older age (aIRR 1.5; 95% CI: 1.2–3.2; *p* = 0.021), reinfection (aIRR 2.0; 95% CI: 1.3–3.0; *p* = 0.001), having positive household contacts (aIRR 2.1; 95% CI: 1.4–3.2; *p* < 0.001) and hospitalisation (aIRR 7.7; 95% CI: 3.8–15.6; *p* < 0.001) increased the risk of developing LC. Post‐infection hospitalisation was significantly associated with symptoms such as anxiety (aIRR 1.4; 95% CI: 1.1–1.7; *p* = 0.009), depression (aIRR 1.9; 95% CI: 1.6–2.3; *p* < 0.001), sore throat (aIRR 1.5; 95% CI: 1.7–2.0; *p* = 0.002) and weight loss (aIRR 1.8; 95% CI: 1.4–2.4; *p* < 0.001). A considerable percentage of participants with post‐SARS‐CoV‐2 infections presented with long‐term complications and required medical intervention. Postpandemic healthcare planning and resource allocation need to be considered since increased morbidities associated with LC place a burden on the already inadequately funded healthcare system.

## 1. Introduction

COVID‐19 had a major impact globally, particularly in South Africa (SA), which recorded 102,595 deaths and 4,076,463 confirmed cases between January 3, 2020, and April 13, 2024 [1]. KwaZulu‐Natal (KZN) in SA experienced a severe impact from COVID‐19, reporting 16,297 deaths and 728,708 confirmed cases by April 2023, making it the province with the second‐highest number of infections in the country [2]. The province has the highest rates of HIV/AIDS and TB in the country and is particularly vulnerable to COVID‐19 due to existing comorbidities that worsen health outcomes [3]. Additionally, KZN has the second‐highest number of informal settlements in SA, following Gauteng [4]. The province consists of 11 districts, with uMgungundlovu and eThekwini Districts having the highest COVID‐19 mortality and morbidity rates. In the eThekwini District, 17.1% of 2.1 million residents live below the poverty line of R1,227 per person per month [5]. In uMgungundlovu, 63.4% of the population lives below the poverty line, with 45.6% lacking any income sources and 17.8% earning under R400 per month [6].

Cities are major sources of COVID‐19 infections due to their dense populations and economic activities [7]. Research indicates that in affluent countries with modern welfare systems, health risks and causes of death are unevenly distributed, with lower socioeconomic status individuals facing higher risks of illness and premature death [8]. Health inequalities exist in chronic diseases, including chronic infections like tuberculosis (TB), and acute infections such as viral respiratory diseases, showing variations in frequency and severity [9, 10]. Recovered COVID‐19 patients are facing persistent symptoms, particularly neurological and psychiatric issues, that can last for weeks or months as the virus evolves with new strains. COVID‐19, while primarily a respiratory infection, is a multisystem illness that can lead to prolonged symptoms known as long COVID‐19 (LC) [11, 12]. LC was defined as ‘an infection‐associated chronic condition that occurs after SARS‐CoV‐2 infection and is present for at least 3 months as a continuous, relapsing and remitting, or progressive disease state that affects one or more organ systems’ [13].

Anyone infected with COVID‐19 can develop LC, with approximately 17 million individuals in the WHO European Region potentially affected in the initial two years of the pandemic (2020–2021) [14, 15]. An analysis of 41 reports estimated a global prevalence of LC of approximately 43% [16], although some studies reported a prevalence of up to 80% among previous patients [17]. Jassat et al. (2022) highlight a high prevalence of LC in SA, with 39% of 3700 participants reporting symptoms 6 months post‐infection. The affected rate was notably higher among hospitalised individuals (46.7%) compared to nonhospitalised participants (18.5%) [18]. Another study in Cape Town, SA, found that 60% of patients with mild COVID‐19 experienced at least one LC symptom, with 35% reporting three or more symptoms persisting for two months [19]. Although the two SA studies looked at the prevalence of LC, there is no much research that focuses on LC in KZN overall or in the uMgungundlovu and eThekwini Districts.

Increased prevalence of LC is noted in severely ill and unvaccinated individuals, complicating the distinction between vaccine‐related adverse events and LC symptoms due to vaccination campaigns [20]. Concerns about the causes and mechanisms of LC have heightened vaccine hesitancy [21]. However, COVID‐19 vaccines may prevent or treat LC by blocking SARS‐CoV‐2 infection, reducing severity in vaccinated individuals, and providing benefits to those already affected by LC [22]. Twelve studies showed that 41.7% of COVID‐19 survivors experienced lingering LC symptoms, with 14.1% unable to return to work two years post‐infection, leading to increased disabilities and a labour shortage [23]. O’Mahoney et al. found that 45% of COVID‐19 survivors had at least one unresolved symptom, averaging 126 days post‐follow‐up, with a higher prevalence in posthospitalised individuals compared to nonhospitalised survivors [11, 24]. Estimates suggest that 10%–30% of nonhospitalised and 50%–70% of hospitalised COVID‐19 cases experience LC, affecting 10%–12% of vaccinated individuals as well [25, 26]. Additionally, reinfection may elevate the risk of developing LC [27].

LC symptoms vary across different conditions such as myalgia, encephalomyelitis, chronic fatigue syndrome, dysautonomia, type 2 diabetes and cardiovascular diseases (CVDs) [12, 15, 16]. Respiratory problems such as dyspnoea and cough impact 20%–40% of people for at least seven months, while gastrointestinal symptoms may manifest as heartburn, nausea, constipation, loss of appetite and abdominal pain [11, 28]. The gastrointestinal and respiratory symptoms may diminish, whereas neurocognitive symptoms may deteriorate over time [29, 30]. Patients with acute SARS‐CoV‐2 infection, particularly those with severe cases requiring hospitalisation, are more likely to face readmission [30]. A 2021 U.K. study reported that 1.6% of individuals faced multiple rehospitalisations, with a median readmission time of 6 days, mainly attributed to COVID‐19 infection, sepsis, pneumonia and heart failure [31].

LC is associated with new cardiovascular conditions in healthy individuals, impacting CVD through symptoms like arrhythmias, ischaemic events, inflammation and cardiac arrest. Patients with LC are 1.6 times more likely to develop new CVD according to a study involving 153,760 patients [32]. The interaction between LC and the cardiovascular system influences its presentation, mechanisms and risk assessment [33]. Patients with LC have autoantibodies such as anticardiolipin and anti‐apolipoprotein A‐1, which are associated with CVD and adverse health outcomes [34]. However, conflict exists about whether pre‐existing cardiac conditions, specifically hypertension, are linked to LC sequelae, as two studies found no connection [35, 36]. Moreover, a meta‐analysis of 10 longitudinal studies in the United Kingdom found that hypertension and hypercholesterolaemia are not significant predictors of LC [37].

Additionally, the COVID‐19 pandemic has heightened concerns regarding a possible rise in undiagnosed chronic fatigue syndrome cases [28, 31, 38, 39]. A study in SA (2022) revealed that fatigue was the most common chronic symptom among nonhospitalised individuals with LC 6 months after acute infection (11.7%), headache (4.9%), shortness of breath (SOB) (4.9%), a persistent cough (3.5%), loss of smell (3.3%) and nasal congestion (3.3%) [18]. Marjenberg et al. (2023) found that COVID‐19 infection significantly increases the risk of fatigue (RR: 1.72), SOB (RR: 2.60), memory problems (RR: 2.53) and concentration problems [40]. LC risk factors include gender, age, ethnicity, comorbidities, a higher frequency of acute COVID‐19 symptoms and severe COVID‐19 disease [15, 18, 41]. Jassat et al. identified risk factors for LC, including older age, females, comorbidities, acute COVID‐19 symptoms and hospitalisation or severity of COVID‐19 [18].

LC poses significant challenges to South Africa’s public health system, particularly in district health clinics facing limited resources and high patient volumes, making diagnosis and treatment more difficult. The initial findings of the review highlight significant barriers faced by district health clinics in diagnosing LC. The healthcare system in SA is evolving, albeit facing challenges, in its ability to prevent and manage LC, with a focus on incorporating care into standard primary health services [42]. During the COVID‐19 pandemic, KZN’s health systems focused on acute care and vaccination efforts, which resulted in a lack of preparedness for LC care services [43]. Currently, the province is integrating LC management into chronic care services, focusing on rehabilitation, telemedicine and community‐based care to alleviate pressure on tertiary hospitals [44].

Self‐isolated COVID‐19 patients are underrepresented in LC research, creating gaps in knowledge regarding symptom prevalence, severity, trajectory and other outcomes. The effects of age, comorbidities, vaccination status, self‐management practices and socioeconomic or regional factors on recovery are not well‐understood. Limited data on the prevalence of LC in self‐isolated patients in SA exists, notwithstanding the Cape Town study’s findings on noncritical COVID‐19 patients, which had a follow‐up of only two months postdiagnosis, indicating a brief observation period [19]. Additionally, while pandemics have historically impacted cities, before COVID‐19, there was limited literature on the relationship between cities and pandemics [7]. Urban research on past pandemics highlights the inequalities that heighten vulnerability for impoverished and marginalised communities [45]. Therefore, our study aimed to assess characteristics associated with post‐COVID‐19 and to describe LC symptoms six months after COVID‐19 diagnosis among individuals who experienced mild symptoms and self‐isolated at home during their initial illness in the two cities of KZN, SA. The study’s findings will guide policymakers on the pandemic’s impacts, risk factors, illness processes, public health strategies and future outbreak protocols to enhance care and well‐being.

## 2. Materials and Methods

### 2.1. Study Design

A cross‐sectional study was conducted among 280 participants who had previously contracted COVID‐19 in the two largest districts (uMgungundlovu and eThekwini Metro Municipality) of KZN Province. Authors adhered to STROBE guidelines for the design of cross‐sectional studies in manuscript preparation.

### 2.2. Study Setting

The province is located in the southeastern part of SA with the second‐highest population and COVID‐19 infection rate in the country. KZN is SA’s epicentre of other morbidities like the human immunodeficiency virus (HIV)/acquired immunodeficiency syndrome (AIDS) epidemic, as well as a high incidence of hospital admissions for drug‐resistant TB [36, 37]. The province has 11 districts; however, the study was conducted in uMgungundlovu District (District A) (which serves as its capital city) and eThekwini Metro Municipality (District B, the largest city). The two districts have the highest number of COVID‐19–related morbidity and mortality. Additionally, eThekwini and uMgungundlovu municipalities in KZN face significant disease burdens, including HIV/AIDS, TB and noncommunicable diseases [46, 47].

### 2.3. Study Population and Sampling Strategy

The study sample included participants aged 18 and older who had tested positive for SARS‐CoV‐2 via reverse transcription polymerase chain reaction (RT‐PCR) or rapid antigen test within the last six months and had self‐isolated at home in Districts A and B. The study excluded COVID‐19 patients from other districts in KZN Province, those in informal settlements unable to self‐isolate and individuals isolated in healthcare facilities. Sample size determination for the study was guided by the COVID‐19 case prevalence in KZN, incorporating a 95% confidence interval and a 5% margin of error. To calculate the sample size (*n*), the formula used was *n* = *Z*2 × *p* (1 − *p*)/(*e*)2, where *Z* was the confidence interval level at 95% (standard value 1.96), *p* was the proportion of KZN cases over the SA cases and *e* was 5% margin of error (range of confidence interval), yielding a total of 283 cases. A convenience sampling strategy was utilised to select patients. Participants were selected using provincial data received daily from the National Institute for Communicable Diseases (NICD).

### 2.4. Data Collection

Participants were identified through a COVID‐19 provincial case line list that supplied essential contact information and basic details needed for recruitment. Prior to participation, both verbal and written informed consent were obtained from all participants to ensure ethical compliance and voluntary participation in the study. Interviews were conducted in participants′ preferred language, either English or isiZulu, to enhance clear communication, participant comfort and reliability of responses while minimising language barriers. The questionnaire was designed to collect comprehensive information related to the objectives of the study. It covered demographic characteristics such as age, gender, employment status and residence, alongside participants’ COVID‐19 vaccination details, including vaccination status, timing relative to infection, number of doses and clinical information. Furthermore, the questionnaire included items regarding long‐term health‐related symptoms experienced by participants. The questions assessing long‐term outcomes were adapted from the Physical Functioning Questionnaire of the National Health and Nutrition Survey to evaluate participants’ functional and health outcomes 6 months after testing positive for COVID‐19. The use of a standardised instrument ensured consistency and reliability in the data collected. Participants who reported their COVID‐19 vaccination status were categorised as fully vaccinated before or after the SARS‐CoV‐2 infection, or unvaccinated.

### 2.5. Data Analysis

Data were cleaned using an Excel spreadsheet, removing patient‐identifying information, and analysed using STATA 17.0. We employed frequency distribution tables and graphs to depict demographics, symptom prevalence and changes in health. Categorical data were summarised using frequencies and percentages, while continuous data were reported using interquartile ranges (IQR) and medians. A modified Poisson regression model was utilised to identify predictors of LC in patients, producing incidence risk ratios (IRR)/adjusted IRR (aIRR) and 95% confidence intervals (95% CI) for LC symptom development. All the variables had variance inflation factor (VIF) values of less than 10. The list of all the predictor variables was drawn first, then the association between each variable and the outcome was examined per variable (unadjusted analysis). A manual forward stepwise method was used to identify the independent predictor variables. We used a *p* value of 0.25 as the cutoff value in the unadjusted analysis for inclusion in the adjusted model, which is supported by the literature [48–50]. In the adjusted analysis, the *p* value < 0.05 was considered statistically significant.

### 2.6. Ethical Considerations

The study adhered to the principles of the Declaration of Helsinki and obtained both verbal and written informed consent from the participants. It received ethical clearance from the Durban University of Technology Institutional Research Ethics Committee (DUT IREC 109/21), with permission from the KZN Department of Health’s Research Ethics Committee and the National Institute for Communicable Diseases (NICD) (under the National Health Laboratory Services).

## 3. Results

### 3.1. Participants’ Baseline Sociodemographic Characteristics

The study included 280 participants; three did not meet the inclusion criteria and were self‐isolating at home during acute COVID‐19 infection. Over 60% (61%, *n* = 170) of the participants were from District B. The median age was 34 years (IQR: 29–47), with females constituting 63% (*n* = 177). Of the participants, 60% (*n* = 164) were full‐time workers. Approximately (70%, *n* = 195) reported having repeated COVID‐19 tests, with 38% (*n* = 74) testing positive for the repeated tests. The majority (85%, *n* = 238) had received vaccinations, with 69% (*n* = 165) receiving them after their initial COVID‐19 infection. Eighty participants (29%) reported that other household members also tested positive during their isolation period. Approximately half of the participants (*n* = 139) experienced at least one hospital admission after their initial COVID‐19 infection due to persistent symptoms. Over half of the participants (55%, *n* = 153) reported that their symptoms persisted 6 months post‐infection (Table 1).

**TABLE 1:** Characteristics of COVID‐19 participants who self‐isolated at home, KwaZulu‐Natal, South Africa (*N* = 280).

Participants		Total^∗^ (*N* = 280)	Percentage (%)
Age (years)	Median (IQR)	34 (29–47)	n/a

Age groups (years)	≤ 35	150	53.6
	36–60	104	37.1
	61+	26	9.3

Sex	Female	177	63.2

Ethnic group	African	214	76.4
	Coloured	14	5.0
	Indian	41	14.6
	White	11	4.0

Employment status	Employed full‐time	164	58.6
	Employed part‐time	46	16.4
	Self‐employed	20	7.4
	Pensioner	11	3.9
	Unemployed	39	13.9

District	District A	110	39.3
	District B	170	60.7

Repeated COVID‐19 test	No	85	30.4
	Yes	195	69.6

Repeated test results	Negative	121	62.0
	Positive	74	38.0

Vaccinated	Before COVID‐19 infection	68	24.3
	After COVID‐19 infection	165	58.9

Positive household contacts	No	194	62.3
	Yes	80	28.6

Long COVID‐19 symptoms	No	127	45.4
	Yes	153	54.6

Hospitalisation post‐COVID‐19	No	137	48.9
	Yes	139	49.6

^∗^There were missing data for some of the variables (repeated test results, vaccination status and positive household contacts).

### 3.2. Description of Symptoms Experienced Post‐COVID‐19 Infection

**FIGURE 1:**
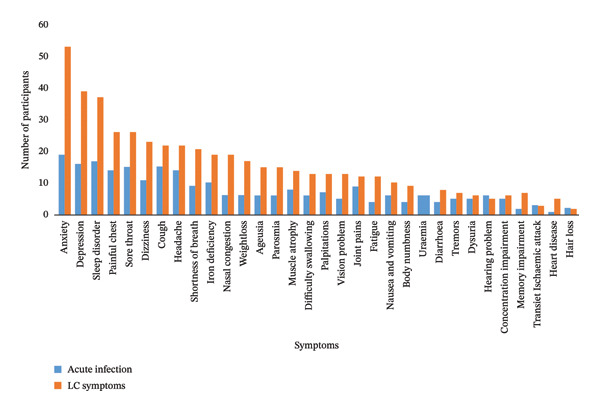
Symptoms experienced at baseline and after 6 months, KwaZulu‐Natal, South Africa.

Six months following diagnosis of COVID‐19, 46% (*n* = 130) of participants experienced at least one COVID‐19–related symptom, with 36% (*n* = 47) experiencing more than five symptoms over an extended period. The most prevalent symptoms included anxiety (58%, *n* = 75), depression (47%, *n* = 61), sleep disorder (45%, *n* = 59), chest pain (35%, *n* = 46) and sore throat (34%, *n* = 44). Other reported LC conditions included hypertension (43%, *n* = 56), iron deficiency (25%, *n* = 32), diabetes mellitus (21%, *n* = 27) and heart diseases (5%, *n* = 6). While 19% (*n* = 25) of the participants reported having palpitations, only 5% (*n* = 6) had a cardiac problem diagnosed. The individuals with rheumatoid arthritis, tremors, memory loss, heart problems and seizures indicated that they developed these symptoms following COVID‐19 infection and vaccination (Figure 1).

### 3.3. LC Symptoms by Age Group and Gender

Using chi‐squared analysis, there was a significant difference in developing hypertension (*p* < 0.001) and losing weight (*p* < 0.001) between different age groups six months after a COVID‐19 infection. However, no significant difference was observed in losing weight (*p* = 0.808) between males and females. Significant differences were observed in the development of persistent headaches among different age groups including persistent headache (*p* = 0.015), dizziness (*p* = 0.001), SOB (*p* = 0.012), nasal congestion (*p* = 0.028), parosmia (*p* = 0.001), ageusia (*p* = 0.002), difficulty swallowing (0.005), heart palpitations (*p* = 0.001), painful joints (*p* < 0.001) and fatigue (*p* = 0.002) (Table 2).

**TABLE 2:** Frequency of individual signs and symptoms by age group and gender, KwaZulu‐Natal, South Africa.

Symptoms		Total (280), *N* (%)	18–35 years, *n* (%)	36–60 years, *n* (%)	> 60 years, *n* (%)	*p* value	Female, *n* (%)	Male, *n* (%)	*p* value
Anxiety	No	205 (73.2)	111 69.2)	72 (84.6)	22 (73.2)	0.271	134 (75.7)	71 (68.9)	0.217
	Yes	75 (26.8)	32 (30.8)	4 (15.4)	75 (26.8)		43 (24.3)	32 (31.1)	

Depression	No	219 (78.2)	84 (80.8)	16 (61.5)	119 (79.3)	0.093	147 (83.1)	72 (69.9)	**0.010**
	Yes	61 (21.8)	20 (19.2)	10 (38.5)	31 (20.7)		30 (19.9)	31 (30.1)	

Sleep disorder	No	221 (78.9)	83 (79.8)	20 (76.9)	118 (78.7)	0.943	142 (80.2)	79 (76.7)	0.485
	Yes	59 (21.1)	21 (20.2)	6 (23.1)	32 (21.3)		35 (19.8)	24 (23.3)	

Hypertension	No	224 (80.0)	77 (74.0)	11 (42.3)	136 (90.7)	**< 0.001**	150 (84.7)	74 (71.8)	**0.009**
	Yes	56 (20.0)	27 (26.0)	15 (57.7)	14 (9.3)		27 (19.3)	29 (28.2)	

Sore throat	No	236 (84.3)	86 (82.7)	20 (76.9)	130 (86.7)	0.386	154 (87.0)	82 (79.6)	0.101
	Yes	44 (15.7)	18 (17.3)	6 (23.1)	20 (13.3)		23 (13.0)	21 (20.1)	

Cough	No	239 (85.4)	133 (88.7)	85 (81.7)	21 (80.8)	0.241	159 (89.8)	80 (77.7)	**0.006**
	Yes	41 (16.4)	17 (11.3)	19,918.3)	5 (19.2)		18 (10.2)	23 (22.3)	

Headache	No	240 (85.7)	135 (90.0)	87 (83.6)	18 (69.2)	**0.015**	156 (88.1)	84 (81.5)	0.129
	Yes	40 (14.3)	15 (10.0)	17 (16.4)	8 (30.8)		21 (11.9)	19 (18.5)	

Dizziness	No	242 (86.4)	139 (92.7)	85 (81.7)	18 (69.2)	**0.001**	157 (88.7)	85 (82.5)	0.146
	Yes	38 (13.6)	11 (7.3)	19 (18.3)	8 (30.8)		20 (11.3)	18 (17.5)	

SOB	No	244 (87.1)	138 (92.0)	87 (83.6)	19 (73.1)	**0.012**	161 (91.0)	83 (80.6)	**0.012**
	Yes	36 (12.9)	12 (8.0)	17 (16.4)	7 (26.9)		16 (9.0)	20 (19.4)	

Nasal congestion	No	251 (89.6)	141 (94.0)	89 (85.6)	21 (80.8)	**0.028**	162 (91.5)	89 (86.4)	0.175
	Yes	29 (10.4)	9 (6.0)	15 (14.4)	5 (19.2)		15 (8.5)	14 (13.6)	

Parosmia^∗^	No	253 (90.4)	144 (96.0)	89 (85.6)	20 (76.9)	**0.001**	163 (92.1)	90 (87.4)	0.198
	Yes	27 (9.6)	6 (4.0)	15 (14.4)	6 (23.1)		14 (7.9)	13 (12.6)	

Weight loss	No	257 (91.8)	146 (97.3)	91 (87.5)	20 (76.9)	**< 0.001**	163 (92.3)	94 (91.3)	0.808
	Yes	23 (8.2)	4 (2.7)	13 (12.5)	6 (23.1)		14 (7.9)	9 (8.7)	

Ageusia^∗^	No	254 (90.7)	144 (96.0)	90 (86.5)	20 (76.9)	**0.002**	164 (92.7)	90 (87.4)	0.142
	Yes	26 (9.3)	6 (4.0)	14 (13.5)	6 (23.1)		13 (7.3)	13 (12.6)	

Difficulty swallowing	No	255 (91.1)	143 (95.3)	92 (88.5)	20 (76.9)	**0.005**	167 (94.3)	88 (85.4)	**0.012**
	Yes	25 (8.9)	7 (94.7)	12 (11.5)	6 (23.1)		10 (5.7)	15 (14.6)	

Palpitations	No	255 (91.1)	148 (96.7)	90 (86.5)	20 (76.9)	**0.001**	164 (92.7)	91 (88.3)	0.223
	Yes	25 (8.9)	5 (3.3)	14 (13.5)	6 (23.1)		13 (7.3)	12 (11.6)	

Joint pains	No	256 (91.4)	146 (97.3)	91 (87.5)	19 (73.1)	**< 0.001**	165 (93.2)	91 (88.3)	0.160
	Yes	24 (8.6)	4 (2.8)	13 (12.5)	7 (26.9)		12 (6.8)	12 (11.7)	

Fatigue	No	259 (92.5)	146 (97.3)	92 (88.5)	21 (80.8)	**0.002**	164 (92.7)	95 (92.2)	0.897
	Yes	21 (7.5)	4 (2.7)	12 (11.5)	5 (19.2)		13 (7.3)	8 (7.8)	

*Note:* The bold values indicate significance with a *p* value threshold of ≤ 0.005.

^∗^SOB: shortness of breath; ageusia: loss of testing sensation; parosmia: loss of smelling sensation; there were missing data for some of the symptoms.

### 3.4. Repeated COVID‐19 Tests Post‐Acute Infection

Participants were required to repeat tests due to persistent COVID‐19 symptoms. Out of 195 individuals who repeated COVID‐19 tests, 38% (*n* = 74) were reinfected. The main reason for not accepting a follow‐up test was the unpleasant swabbing experience (63%, *n* = 24). However, some symptomatic participants visited health facilities but were not offered tests (24%, *n* = 9) (Figure 2).

FIGURE 2Frequency and reasons for failed follow‐up COVID‐19 tests, KwaZulu‐Natal, South Africa. (a) Frequency of repeated tests. (b) Reasons for not repeating the test.

**Figure:**
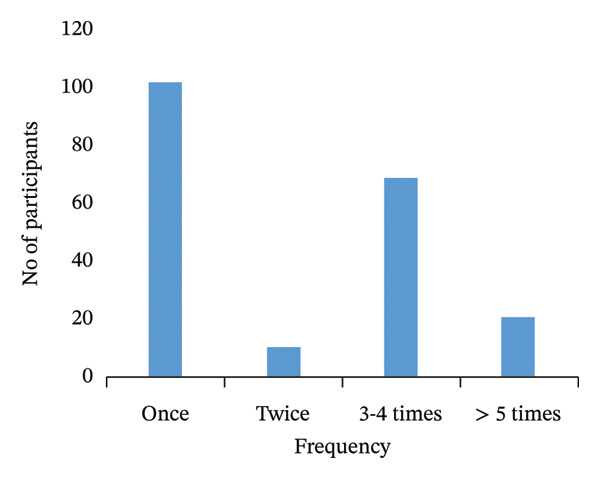
(a)

**Figure:**
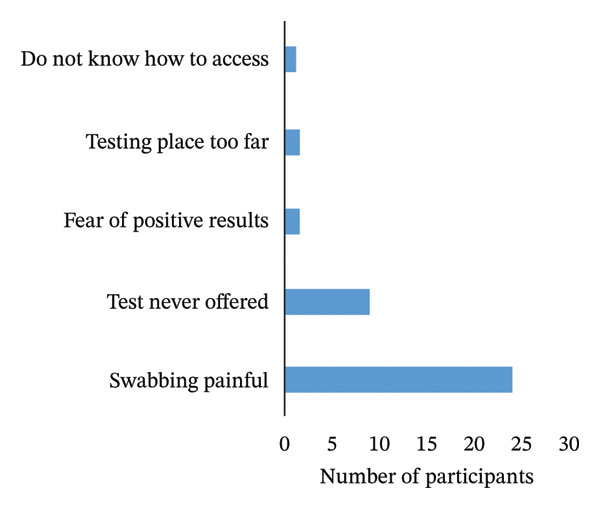
(b)

### 3.5. Characteristics of Participants by Hospitalisation Status (Hospitalisation due to Persistent Symptoms)

The participants who were hospitalised were older than those who never required hospitalisation due to persistent symptoms (median 38 [28–37, 39–51] vs. 32 [27–39, 52]). Females contributed 58% (*n* = 81) of those who required hospitalisation post‐infection. Above 70% (*n* = 87) of those who received vaccination after contracting COVID‐19 were hospitalised. Among pensioners who self‐isolated, the majority (73%, *n* = 8) had at least one hospitalisation post‐infection. Using chi‐squared analysis, there was a significant difference among different age groups (*p* < 0.001); residing in different districts (*p* = 0.034); repeating the test (*p* < 0.001); reinfection (*p* = 0.001); having other household members (*p* < 0.001); and frequency of symptoms (*p* < 0.001) in those who required hospitalisation post‐infection (Table 3).

**TABLE 3:** Characteristics of participants by hospitalisation status, KwaZulu‐Natal, South Africa.

Participants		Total, *N* (%)	Nonhospitalised, *n* (%)	Hospitalised, *n* (%)	*p* value
Age (years)	Median (IQR)	34 (29–47)	32 (27–40)	38 (30–52)	

Age groups (years)	≤ 35	149 (54.0)	90 (65.7)	59 (42.4)	**< 0.001**
	36–60	101 (36.6)	40 (29.2)	61 (43.9)	
	61+	26 (9.4)	7 (5.1)	19 (13.7)	

Sex	Female	174 (63.0)	93 (67.9)	81 (58.3)	0.098

Ethnic group	African	211 (76.4)	106 (77.4)	105 (75.5)	0.457
	Coloured	14 (5.1)	8 (5.8)	6 (4.3)	
	Indian	40 (14.5)	20 (14.6)	20 (14.4)	
	White	11 (4.0)	3 (2.2)	8 (5.8)	

Employment status	Employed full‐time	161 (58.3)	86 (62.8)	75 (54.0)	0.069
	Employed part‐time	46 (16.7)	25 (18.2)	21 (15.1)	
	Self‐employed	20 (7.2)	11 (8.0)	9 (6.50	
	Pensioner	11 (4.0)	3 (2.2)	8 (5.8)	
	Unemployed	38 (13.8)	12 (8.8)	26 (18.7)	

District	District A	108 (39.1)	45 (32.8)	63 (45.3)	**0.034**
	District B	168 (60.9)	92 (67.1)	76 (54.7)	

Repeated COVID test	No	82 (29.7)	55 (40.1)	27 (19.4)	**< 0.001**
	Yes	194 (70.3)	82 (59.8)	112 (80.6)	

Repeat test results	Negative	120 (43.5)	52 (37.9)	68 (48.9)	**0.001**
	Positive	74 (26.8)	30 (21.9)	44 (31.6)	

Positive household contacts	No	190 (68.8)	111 (81.0)	79 (56.8)	**< 0.001**
	Yes	80 (29.0)	22 (2.9)	58 (41.7)	

Vaccination timing	After infection	165 (59.8)	78 (56.9)	87 (62.6)	0.625
	Before infection	67 (24.3)	36 (26.3)	31 (22.3)	

Frequency of LC symptoms	Once	33 (12.0)	10 (7.3)	23 (16.5)	**< 0.001**
	Twice	26 (9.4)	7 (5.1)	19 (13.7)	
	3–4 times	33 (12.0)	16 (11.7)	17 (12.3)	
	> 5 times	37 (13.4)	13 (9.5)	24 (17.3)	

*Note:* The bold values indicate significance with a *p* value threshold of ≤ 0.005.

### 3.6. Persistent Symptoms Associated With Hospitalisation of Patients Post‐Infection

Cough (49%, *n* = 68), fever (44%, *n* = 61), chest pain (42%, *n* = 59), SOB (42%, *n* = 58), loss or change in smelling sensation (42%, *n* = 58) and difficulty swallowing (41%, *n* = 57) were the most common symptoms experienced by those who needed hospitalisation. Post‐infection hospitalisation was significantly associated with symptoms such as anxiety (aIRR 1.4; 95% CI: 1.1–1.7; *p* = 0.009), depression (aIRR 1.9; 95% CI: 1.6–2.3; *p* < 0.001), sore throat (aIRR 1.5; 95% CI: 1.7–2.0; *p* = 0.002) and weight loss (aIRR 1.8; 95% CI: 1.4–2.4; *p* < 0.001) (Table 4).

**TABLE 4:** Symptoms associated with hospitalisation of patients living with long COVID‐19 syndrome, KwaZulu‐Natal, South Africa.

Symptoms		Total *N* = 280	Nonhospitalised, *n* (%)	Hospitalised, *n* (%)	Unadjusted IRR: 95% CI, *p* value	Adjusted aIRR 95% CI, *p* value
Anxiety	No	201 (72.8)	122 (89.0)	79 (56.8)	Ref^∗^	
	Yes	60 (43.2)	15 (11.0)	60 (43.2)	2.0 (1.7–2.5); < 0.001	1.4 (1.1–1.7); **0.009**

Depression	No	215 (77.9)	137 (100.0)	78 (56.1)	Ref^∗^	
	Yes	61 (22.1)	0 (0.0)	61 (43.9)	2.8 (2.3–3.3); **< 0.001**	1.9 (1.6–2.3); **< 0.001**

Sleep disorder	No	217 (78.6)	126 (92.0)	91 (65.5)	Ref^∗^	
	Yes	59 (21.4)	11 (8.0)	48 (34.5)	1.9 (1.6–2.4); **< 0.001**	

Sore throat	No	236 (84.3)	137 (59.0)	0 (0.0)	Ref^∗^	
	Yes	44 (15.7)	95 (41.0)	44 (100.0)	2.4 (2.1–2.8); **< 0.001**	1.5 (1.7–2.0); **0.002**

Cough	No	239 (85.4)	137 (58.3)	0 (0.0)	Ref^∗^	
	Yes	41 (16.4)	98 (41.7)	41 (100.0)	2.4 (2.1–2.8); **< 0.001**	

Headache	No	240 (85.7)	137 (58.0)	0 (0.0)	Ref^∗^	
	Yes	40 (14.3)	99 (42.0)	40 (100.0)	2.4 (2.0–2.8); **< 0.001**	

Dizziness	No	242 (86.4)	137 (57.6)	0 (0.0)	Ref^∗^	
	Yes	38 (13.6)	101 (42.4)	38 (100.0)	2.3 (2.0–2.7); **< 0.001**	

SOB	No	244 (87.1)	137 (57.1)	0 (0.0)	Ref^∗^	
	Yes	36 (12.9)	103 (42.9)	36 (100.0)	2.3 (2.0–2.7); **< 0.001**	

Nasal congestion	No	251 (89.6)	137 (55.5)	0 (0.0)	Ref^∗^	
	Yes	29 (10.4)	110 (44.5)	36 (100.0)	2.2 (1.9–2.6); **< 0.001**	

Parosmia	No	253 (90.4)	137 (55.0)	0 (0.0)	Ref^∗^	
	Yes	27 (9.6)	112 (45.0)	27 (100.0)	2.2 (1.9–2.5); **< 0.001**	

Weight loss	No	257 (91.8)	137 (54.1)	0 (0.0)	Ref^∗^	
	Yes	23 (8.2)	116 (48.9)	23 (100.0)	2.2 (1.9–2.5); **< 0.001**	1.8 (1.4–2.4); **< 0.001**

Ageusia	No	254 (90.7)	137 (54.8)	0 (0.0)	Ref^∗^	
	Yes	26 (9.3)	113 (45.2)	26 (100.0)	2.2 (1.9–2.5); **< 0.001**	

Difficulty swallowing	No	255 (91.1)	137 (54.6)	0 (0.0)	Ref^∗^	
	Yes	25 (8.9)	114 (45.4)	25 (100.0)	2.2 (1.9–2.5); **< 0.001**	

Palpitations	No	255 (91.1)	137 (54.6)	0 (0.0)	Ref^∗^	
	Yes	25 (8.9)	114 (45.4)	25 (100.0)	2.2 (1.9–2.5); **< 0.001**	

Joint pains	No	256 (91.4)	137 (54.4)	0 (0.0)	Ref^∗^	
	Yes	24 (8.6)	115 (45.6)	24 (100.0)	2.2 (1.9–2.5); **< 0.001**	0.6 (0.4–0.7); **< 0.001**

Fatigue	No	259 (92.5)	137 (53.7)	0 (0.0)	Ref^∗^	
	Yes	21 (7.5)	118 (46.3)	21 (100.0)	2.2 (1.9–2.5); **< 0.001**	

^∗^SOB: shortness of breath; all the symptoms had some missing observations; Ref: reference.

### 3.7. Factors Associated With the Development of LC Syndrome

Bivariate analysis showed a significant association between the development of LC and age (*p* < 0.001); those who had to repeat the test due to recurring symptoms (*p* < 0.001); having positive household contacts (*p* < 0.001); the district the participants lived in (*p* < 0.001); the presence of symptoms (*p* < 0.001), reinfections (*p* = 0.001) and hospitalisation (*p* < 0.001). Adjusted analysis indicated that older age (aIRR 1.5; 95% CI: 1.2–3.2; *p* = 0.021), reinfection (aIRR 2.0; 95% CI: 1.3–3.0; *p* = 0.001), having positive household contacts (aIRR 2.1; 95% CI: 1.4–3.2; *p* < 0.001) and hospitalisation (aIRR 7.7; 95% CI: 3.8–15.6; *p* < 0.001) increased the risk of developing LC. This study found no significant correlation between persistent symptoms and age, sex, ethnicity, or self‐reported vaccination status before or after SARS‐CoV‐2 infection (Table 5).

**TABLE 5:** Determinants of long COVID among participants self‐isolating at home, KwaZulu‐Natal, South Africa.

Variable		Unadjusted analysis		Adjusted analysis	
		IRR (95% CI)	*p* value	aIRR (95% CI)	*p* value
Age in years	≤ 35	Ref^∗^			
	36–60	1.4 (1.0–1.8)	**0.027**		
	61+	1.0 (0.5–1.7)	0.964	1.5 (1.1–2.1)	**0.021**

Sex	Female	Ref^∗^			
	Male	1.1 (0.8–1.4)	0.531		

District	District B	Ref^∗^			
	District A	1.2 (0.9–1.6)	**0.107**		

Ethnic group	Indian	Ref^∗^			
	African	1.5 (0.9–2.6)	0.086		
	Coloured	1.2 (0.5–2.8)	0.646		
	White	2.2 (1.1–4.2)	**0.020**		

Type of employment	Unemployed	Ref^∗^			
	Employed full‐time	1.3 (0.7–2.7)	0.384		
	Employed part‐time	1.5 (0.7–3.2)	0.267		
	Pensioner	1.2 (0.4–3.4)	0.715		

Reinfected	No	Ref^∗^			
	Yes	1.9 (1.4–2.6)	**< 0.001**	2.0 (1.3–3.0)	**0.001**

Positive household contacts	No	Ref^∗^			
	Yes	1.8 (1.4–2.4)	**< 0.001**	2.1 (1.4–3.2)	**0.001**

Hospitalisation post‐COVID‐19	No	Ref^∗^			
	Yes	3.4 (2.4–4.9)	**< 0.001**	7.7 (3.8–15.6)	**< 0.001**

Vaccinated	No	Ref^∗^			
	Yes	0.8 (0.5–1.2)	0.274		

Vaccination timing	Before infection	Ref^∗^			
	After infection	1.1 (0.8–1.4)	0.632		

*Note:* The bold values indicate significance with a *p* value threshold of ≤ 0.005.

^∗^Ref: reference.

## 4. Discussion

This study investigated determinants for long‐COVID‐19 and hospitalisation post‐infection in patients who self‐isolated at home after diagnosis in KwaZulu‐Natal Province, South Africa. Persistent LC symptoms include anxiety, depression, sleep disorder, chest pain, sore throat and cough. The risk factors for developing LC include older age, positive household contacts, reinfection and hospitalisation post‐COVID‐19 infection. Post‐infection hospitalisation was significantly associated with symptoms such as anxiety, depression, sore throat and weight loss.

Over half of the participants reported persistent symptoms six months after contracting the virus compared to their baseline COVID‐19 symptoms. Consistent with our findings, Taquet et al. found that 57.0% of COVID‐19 survivors had at least one LC symptom throughout the 6 months, while 36.5% had symptoms for the 3 months post‐infection [31]. Other studies show that within 3–6 months after COVID‐19 infection, one in three individuals experienced one or more LC symptoms [18, 51, 53]. In contrast, Richard et al. (2023) reported that 39.7% of participants had persistent LC symptoms for 28 days or more, with 50% experiencing symptoms for 90 days or longer, and 9.8% continuing to experience symptoms after 6 months [53]. The published data are lower than the above‐stated scholars’, possibly due to variations in population characteristics. Persistent symptoms also decreased between 3 and 6 months in a Chinese cohort, from 51% to 40%, and in a French cohort, from 68% to 60% [54]. Other studies with longer follow‐up periods have also shown significant declines at 12 and 24 months [55, 56].

Moreover, the proportion of LC symptoms in our study was also higher than that found in a survey conducted in SA (2023). Though the SA study found a lower proportion of LC symptoms at 6‐month follow‐up, the proportion of persistent symptoms was higher at 3 months (53.5%, *p* ≤ 0.001) [18]. Their findings were comparable to estimates in Italy (34%), China (40%), Switzerland (34%), Saudi Arabia (48%), Russia (50%), the United States (57%), France (60%) and Norway (61%) [11, 30, 53, 57, 58]. Due to ongoing symptoms or new complaints, people with COVID‐19 use healthcare resources more frequently [59]. Valdivieso‐Martínez et al. found an excess healthcare burden for patients 6 months after acute infection, leading to increased hospitalisations and emergency department visits in a Canadian study [60]. LC varies significantly among individuals due to factors like pre‐existing health conditions, infection severity and specific symptoms experienced.

In this study, the most common LC symptoms after six months of COVID‐19 infection included anxiety, insomnia, depression, sleep disorder, headache, unusual dizziness, fatigue and weight loss. Our findings align with previous LC studies, identifying fatigue, sleep disorder, weight loss, headache, ageusia and anosmia as prevalent symptoms [18, 53, 61]. Boggero et al. found that the most frequent LC symptoms were arthralgia or myalgia, sleeplessness, melancholy, anxiety and exhaustion [62]. The review of LC studies following COVID‐19 reveals deteriorated mobility, self‐care, regular activities, pain, discomfort, anxiety and depression, leading to a loss of independence [62, 63]. In addition to symptoms and complications, LC patients often experience impaired quality of life, mental health issues and employment issues [64].

While LC symptoms rarely require hospitalisation, severe symptoms such as respiratory distress, chest pain, high fever, neurological issues, fatigue, mental health problems and gastrointestinal issues may necessitate hospitalisation [65]. Our study found that 82% of LC patients required hospitalisation (indicating severity) within six months post‐COVID‐19 diagnosis due to persistent symptoms, highlighting the virus’s significant impact on quality of life. Severe LC symptoms correlate with a heightened immune response and cytokine storm, leading to increased organ damage and more stringent treatment requirements [18, 66]. Moreover, our study aligns with previous research, identifying common symptoms in hospitalised LC patients such as cough, fever, chest pain, depression, SOB, sore throat, loss of taste, sensation and difficulty swallowing [67, 68].

Additionally, in this study, post‐infection hospitalisation was found to be significantly linked to symptoms like anxiety, depression, sore throat and weight loss. A trial involving 634,734 people found a significant correlation between anxiety, depression and LC symptoms in individuals (odds ratio, 1.19; 95% CI, 1.02 to 1.40; *I* ^2^ = 96%) [53]. A survey conducted in Malaysia (2022) also found that one in five respondents experienced stress, anxiety and depression due to the consensus on prolonged recuperation [51]. The Malaysian study found that the psychiatric effects of COVID‐19 are influenced by several factors, such as the virus itself, cerebrovascular illness, physiological compromise, immune system responses, medical interventions, social isolation and stigma [69]. Additionally, Badinlou et al. identified a correlation between severe COVID‐19 infection, hospitalisation, and LC impairments and fatigue with increased levels of depression, anxiety and insomnia [70].

Our study found a correlation between older age (≥ 61 years) and the development of LC. Consistently, studies indicate that older age is a significant risk factor for LC [71, 72]. A study of 65+ COVID‐19 patients found that 95% had LC, with fatigue, cough, and breathlessness as the most common symptoms after 90 days [71]. A systematic review conducted by Tsampasian et al. found that, in comparison to younger patients, those 70 years of age or older and those between the ages of 40 and 69 had an equally high risk of LC symptoms [73]. The review highlights that LC prevalence is higher in individuals who have survived the acute COVID‐19 infection, with older individuals less likely to survive due to increased risk of severe illness [73]. This finding was consistent with the results from studies that found a higher prevalence of LC among younger age groups [74]. However, in contrast, other studies found that the increase in LC risk was cross‐cutting for all age groups, indicated by studies globally [15, 57, 73].

Our study found that the severity of the disease (indicated by hospitalisation) increased the risk of developing LC. Previous investigations have also revealed that a substantial risk factor for LC is severe sickness. A meta‐analysis revealed that patients who were hospitalised or admitted to the intensive care unit had more than double the risk of developing LC [74]. In our study, the risk of developing LC among those who had severe LC symptoms was eight times higher than that of those who never required hospitalisation (*p* < 0.001). A U.K. study revealed 20% of COVID‐19 patients did not return to work within five months, while 55% had to change jobs due to health issues [75]. Supporting the findings, Krysa et al. found that hospitalised LC respondents reported more limitations on daily activities and difficulty returning to work compared to nonhospitalised respondents [76].

This study also found that over 80% of individuals received vaccinations, but 69% indicated that they had received their shots after contracting COVID‐19. COVID‐19 vaccination reduces the risk of developing LC by approximately 27% in adults fully vaccinated before infection, according to the European Centre for Disease Prevention and Control [77]. A U.K. study also indicated that vaccination after infection significantly reduces LC symptoms, with further enhancements observed after the second dose [78]. Additionally, Liu et al. indicated that LC outcomes are inversely related to the number of COVID vaccination doses received, with odds ratios indicating a decreasing risk: one dose (OR 0.77), two doses (OR 0.73), three doses (OR 0.64) and four doses (OR 0.29) [79]. In contrast, Asadi‐Pooya et al. found that receiving a COVID vaccination is associated with prolonged LC symptoms that can last over a year in individuals who were vaccinated after previously contracting COVID‐19 [80]. However, our study found no difference in LC symptoms between vaccinated and unvaccinated participants but did not provide specific information on infection temporality, vaccination type, or immunity proof. Therefore, vaccines are recommended for everyone, including those who have had COVID‐19, as they reduce the risk of severe illness and reinfection.

Lastly, this study also found that having positive household contact and reinfection significantly increased the risk of developing LC. The finding was consistent with a study conducted by Murugesan et al., indicating that household contacts are at an increased risk of acquiring COVID‐19 infection. In turn, positive household contacts increase the risk of reinfection to an index case [81]. In our study, in an unadjusted analysis, we found that having positive household contacts increases the risk of developing LC. Approximately 46% of those who had positive household contacts were reinfected. Moreover, our study found that consistently testing positive raises the risk of LC in our adjusted analysis. The reinfections might be driven by having household contacts test positive. This may be the result of several factors, including sharing items and restrooms, extended exposure in enclosed areas and not wearing masks at home [81]. Reinfections seem to raise the risk of LC, but not as much if they are mild or asymptomatic, as in children and teenagers [82]. A study conducted by Bowe et al. over 2 years revealed that patients who experienced reinfection had a higher chance of developing LC [83].

## 5. Limitations

This study has several limitations. In our study, there were no objective metrics to assess symptoms, which might have impacted the reporting. However, we incorporated standardised assessment tools from different validated tools during questionnaire design to address the lack of objective metrics and potential reporting bias. Therefore, future research should concentrate on creating objective assessments and analysing their relationship to self‐reported measures. The study did not gather data regarding the duration of symptoms or the course of therapy after an acute COVID infection, all of which could have an impact on the prevalence of LC. Additionally, participants may have reported health problems more frequently as a result of their heightened awareness of the post‐COVID‐19 syndrome. Moreover, the study only followed up with the participants after 6 months, which might have contributed to the lack of comparison of LC in different time intervals. However, the advantage is that the COVID‐19 diagnosis was laboratory‐confirmed and not based on self‐reports. A key strength of this study is its focus on the enduring effects of LC and the need for continued healthcare support, highlighting the significant efforts required to manage long‐term health risks following the COVID‐19 pandemic, which offers valuable insights for future public health planning.

## 6. Conclusions

Our study analysis indicated that many people infected with SARS‐CoV‐2 had longer‐term effects and a pertinent need for healthcare services. Even after the COVID‐19 pandemic has ended, significant effort will still be required to mitigate the long‐term risks asssociated with COVID‐19 infections.

## Author Contributions

M.P.P. and P.R. were involved in the conception and design of the study. M.P.P. contributed to data collection, initial cleaning, data analysis and interpretation of results. P.R. and A.I. contributed to data interpretation and revision of the manuscript.

## Funding

Partial financial support was received from the Health and Welfare Sector Education and Training Authority (HWSETA) of Health and DUT funded this study.

## Disclosure

The study’s funders did not plan the investigation, gather, analyse, interpret, or write the report. All the authors revised the manuscript and approved the final version.

## Conflicts of Interest

The authors declare no conflicts of interest.

## Data Availability

These data are not publicly available due to the restrictions that apply to their distribution. Nonetheless, upon justifiable request and with approval from the NICD and the South African Department of Health, data can be obtained from the corresponding author.
